# Anti-Phospholipid Antibodies in Women with Placenta-Mediated Complications Delivered at >34 Weeks of Gestation

**DOI:** 10.3390/jcm12134516

**Published:** 2023-07-06

**Authors:** Uri Amikam, Alyssa Hochberg, Michael Shenhav, Lilian Haj, Sarit Hochberg-Klein, Liran Hiersch, Yariv Yogev

**Affiliations:** 1 Lis Hospital for Women, Sourasky Medical Center, Tel Aviv 6423906, Israel; uriamikam@gmail.com (U.A.); shenhav.michael@gmail.com (M.S.); yarivy@tlvmc.gov.il (Y.Y.); 2 Sackler Faculty of Medicine, Tel Aviv University, Tel Aviv 6423906, Israel; alyssahoch@gmail.com (A.H.); lilian.haj@gmail.com (L.H.); 3 Helen Schneider Hospital for Women, Rabin Medical Center, Petach Tikva 4941492, Israel; 4 Palmerston North Hospital, Te Whatu Ora Health NZ, Palmerston North 4442, New Zealand; sarit.hochberg-klein@midcentraldhb.go

**Keywords:** antiphospholipid antibodies, placental-mediated complications, small-for-gestational-age, placental abruption, preeclampsia

## Abstract

Objective: To determine the prevalence of positive antiphospholipid (aPL) antibodies among pregnant women with placenta-mediated complications delivered at >34^0/7^ weeks of gestation. Methods: This was a single-center retrospective observational study conducted between 2017 and 2022. Inclusion criteria included pregnant or post-partum women, >18 years, diagnosed with any of the following placenta-mediated complications and delivered at >34^0/7^ weeks of gestation: small-for-gestational-age neonate (SGA ≤ 5th percentile according to local birthweight charts), preeclampsia with severe features, and placental abruption. The primary outcome was the prevalence of positive aPL antibodies: Lupus anticoagulant, Anticardiolipin, or Anti-ß2glycoprotein1. Results: Overall, 431 women met the inclusion criteria. Of them, 378(87.7%) had an SGA neonate, 30 had preeclampsia with severe features (7%), 23 had placental abruption (5.3%), and 21 patients had multiple diagnoses(4.9%). The prevalence of aPL antibodies in the cohort was 4.9% and was comparable between the three subgroups (SGA—3.9%; PET with severe features—3.3%; and placental abruption—13% (*p* = 0.17)). Conclusion: aPL antibodies prevalence in women with placenta-mediated complications > 34 weeks of gestation was 4.9%, with comparable prevalence rates among the three subgroups. Future prospective studies are needed to delineate the need for treatment in those who tested positive for aPL antibodies and do not meet Anti-Phospholipid Antibody Syndrome clinical criteria.

## 1. Introduction

Antiphospholipid syndrome (APS) is an autoimmune multisystem disorder characterized by venous, arterial, or small vessel thromboembolic events and/or adverse pregnancy outcomes in the presence of persistent laboratory evidence of antiphospholipid (aPL) antibodies [1].

The first Sapporo classification criteria for APS diagnosis was published in 1999 [2] and was revised in 2006 at a consensus workshop in Sydney, Australia [3]. Whereas the Sydney criteria were not designed for clinical purposes, they represent the best available tool for APS diagnosis in clinical practice [4]. In 2013, novel clinical criteria were proposed in order to distinguish between two different entities, that is, thrombotic APS (TAPS) and APS associated with obstetric morbidity (OAPS) [5]. The placental pathophysiology in OAPS includes placental infarction, decidual inflammation, impaired spiral artery remodeling, increased number of syncytial knots, deposition of complement split product C4d, and obliterative arteriopathy [6,7]. While these findings are not specific to OABS, they are associated with pregnancy complications [7].

The three aPL antibodies tests that are recognized by international classification criteria for APS [8] are (1) Anticardiolipin antibodies (aCL) immunoglobulin G (IgG), and/or IgM enzyme-linked immunosorbent assay (ELISA); (2) Anti-β2-glycoprotein-I (β2GPI) antibodies IgG and/or IgM ELISA; (3) and Lupus anticoagulant (LAC) test. The clinical criteria are either a thromboembolic event or pregnancy complications. Obstetrical complications defined as OAPS include either recurrent first-trimester miscarriage, fetal losses, stillbirth, early preeclampsia (PET) with severe features (<34 weeks), or prematurity (<34 weeks) due to placental dysfunction [3].

Over the past years, there has been growing evidence of extra clinical and laboratory manifestations of APS not meeting the strict Sydney criteria [4,9,10,11,12,13]. Furthermore, the 16th international congress on aPL task force [14] called for additional studies to clarify and define the relationship between myriad pregnancy complications and aPL antibodies.

Data in the medical literature regarding APS-related obstetrical complications not meeting the Sydney criteria are scarce, and the studies that do exist are lacking in several aspects, such as testing only part of the aPL antibodies [11,15]; including a small number of cases ranging between 100 and 148 [11,15,16]; cases with no obvious placental pathology such as late preterm deliveries between 34 and 37 weeks with no apparent cause, and recurrent implantation failure [13]. In addition, a mixture of obstetrical complications only partially meeting the Sydney criteria was included [15,16].

Hence, our study aimed to examine the prevalence of aPL antibodies in a predefined group of patients diagnosed with placenta-mediated complications and who delivered at >34^0/7^ weeks of gestation.

## 2. Methods

We conducted an observational retrospective study of all patients who were diagnosed with placenta-mediated complications and delivered after 34 weeks of gestation at Tel Aviv Sourasky Medical Center, a university-affiliated tertiary medical center, between 2017 and 2022.

Gestational age (GA) was determined according to the last menstrual period (LMP) and a first-trimester ultrasound exam. LMP was used to establish the estimated due date (EDD) and was considered consistent with ultrasound dating if the dates were within four days prior to 10^0/7^ weeks, within six days from 10^0/7^–13^6^^/7^ weeks, and within nine days from 14^0/7^ weeks–20^0^^/7^ weeks. If the ultrasound assessment of EDD was not consistent with the LMP, the EDD was based on the ultrasound assessment.

Predefined placental-mediated complications included one or more of the following, diagnosed at any time during gestation, with the delivery occurring after 34 weeks of gestation: SGA (birthweight ≤ 5th percentile according to local birthweight percentiles [17]); placental abruption (confirmed by placental pathology); and PET with severe features which was defined according to the ACOG criteria [18].

Fresh blood was drawn from patients during admission before delivery or in the immediate postpartum period (<48 h after delivery). Screening assays were used to detect aPL antibodies according to the Sydney recommendations of the International Society on Thrombosis and Hemostasis Subcommittee [3,19]. Plasma aCL IgG/IgM and anti-β2GPI IgG/IgM antibody titers were determined by commercial ELISA methods (Orgentec Diagnostika, Mainz, Germany). The results of aCL and anti-β2GPI IgG/IgM were expressed as IgG phospholipid (GPL) or IgM phospholipid (MPL) using international reference material. The cut-off values used for high titers of aCL and anti-β2GPI were in the 99th percentile according to the manufacturer’s recommendations, obtained by testing an age-matched healthy population. The cutoff value for positivity was defined following the manufacturer’s instructions (>10 U/mL and >8 U/mL for aCL and anti-β2GPI IgG/IgM, respectively). Positive titers were further subdivided into two categories: low titer (<40 U/mL), and high titer (>40 U/mL) [20]. The LAC test was performed according to the latest Guidance from the Scientific and Standardization Committee for lupus anticoagulant/antiphospholipid antibodies of the International Society on Thrombosis and Haemostasis [19]. We performed a three-step procedure with two test systems (diluted Russell’s viper venom time and activated partial thromboplastin time (aPTT)). Silica was used as an activator in the aPTT assays. If a patient was treated with low molecular heparin (LMWH), the blood was withdrawn before administering the next dose.

Exclusion criteria included: OAPS and TAPS meeting the Sydney criteria; aPL antibodies testing not done at our institution or previously done in the current pregnancy; abnormal fetal genetic testing or suspected congenital anomalies on ultrasound; active viral infections such as hepatitis B (HBV), hepatitis C (HCV), or human immunodeficiency virus (HIV); and delivery occurring at other institutions.

Medical records of all women who met the inclusion criteria and completed aPL antibodies tests were reviewed. Patients’ data were anonymized and de-identified before analysis. The study was approved by the local institutional review board (IRB TLV-0365-21).

Our primary outcome was to evaluate the presence of aPL antibodies in the study population. Our secondary outcomes were a subgroup analysis comparing the antibodies’ prevalence between the three subgroups of women with placenta-mediated complications (SGA, placental abruption, PET with severe features); between patients receiving LMWH treatment to those not receiving it; and between patients with placental complications occurring in the late preterm (34–36^6/7^ weeks of gestation) versus term pregnancies (37 weeks and above).

Maternal characteristics, obstetrical history, antenatal and intrapartum events, as well as maternal and neonatal outcomes, were reviewed, using hard-copy and electronic medical records.

Statistical analysis was performed using SPSS 25.0 (IBM Corporation, Chicago, IL, USA) software. Continuous data were expressed as mean ± SD, and categorical data were expressed as numbers and percentages. To test the statistical significance, a Chi-Square test was used for categorical variables. The level of significance was set at *p* < 0.05.

## 3. Results

**Figure 1:**
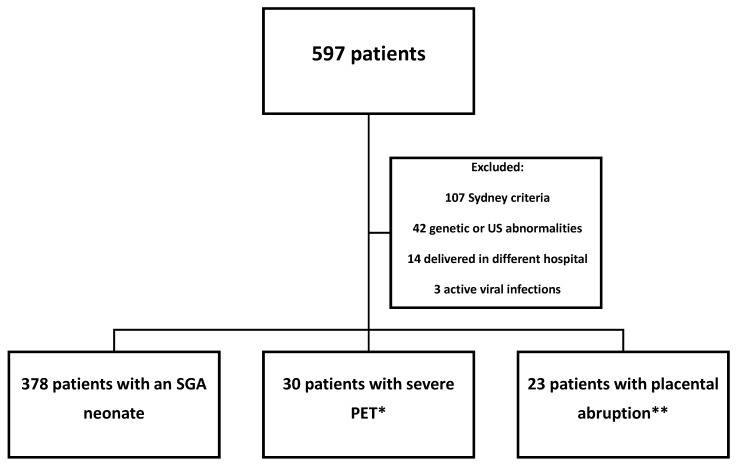
Flow chart of the study population. * 16 patients had multiple classifications. ** 5 patients had multiple classifications. Abbreviations: US—ultrasound; SGA—small-for-gestational-age; PET—preeclampsia.

During the study period, 597 parturients underwent aPL antibodies testing. After implementing exclusion criteria, 431 patients were eligible for analysis (Figure 1).

Patient characteristics are shown in Table 1. Six patients (1.4%) had autoimmune diseases, and none of them had systemic lupus erythematosus (SLE). The most common co-morbidity in our cohort was hypothyroidism (*n* = 21, 4.9%). Eight patients (1.9%) had a diagnosis of inherited thrombophilia.

Pregnancy and delivery characteristics are presented in Table 2. The mean GA at delivery was 38 weeks and 3 days, with 68 patients (15.8%) delivering in the late preterm period. 64 patients (14.9%) conceived via in-vitro fertilization, and there were 11 twin pregnancies (2.6%). During pregnancy, 40 patients (9.3%) were treated with aspirin. More than half of the patients in our cohort (*n* = 225 patients, 52.2%) had fetal genetic testing during their pregnancy which was normal for all. Of them, 182 patients (42.2%) had invasive testing, and 43 (10%) had prenatal cell-free DNA.

Regarding mode of delivery, 255 (59.2%) patients had a vaginal delivery, while 176 (40.8%) had a cesarean delivery (CD). Mean neonatal birthweight was 2289.6 (±324.1) grams, and 407 neonates (92.1%) were diagnosed as SGA (≤10th percentile according to local birthweight charts). Additionally, 331 (74.9%) neonates had a low birthweight, at <2500 g, with 78 (17.7%) neonates born at <2000 g.

Regarding placenta-mediated complications, 378 patients were included in the SGA group, 30 patients in the severe PET group, and 23 patients in the placental abruption group. Of note, there were 16 patients with an SGA neonate who also were diagnosed with PET with severe features, and they were included only in the PET group, and five patients who had a placental abruption and an SGA neonate, who were included only in the placental abruption group. Only one of the patients who had multiple complications had a positive test for aPL antibodies (a patient who suffered from PET with severe features and had an SGA neonate).

The prevalence of aPL antibodies is shown in Table 3. Twenty-one patients (4.9%) in our cohort had a positive aPL antibody test, with five patients (1.2%) having more than one positive antibody and only one patient (0.2%) having triple-positive aPL antibodies testing. While testing for aPL antibodies, 281 patients (65.2%) were treated with LMWH, and there were no patients treated with Warfarin (not shown in the tables). The antibody positivity distribution was as follows: 14 patients were positive for LAC (3.2%), six patients were positive for aCL (1.4%), and six patients were positive for anti-β2GPI (1.4%). Only two patients in the cohort had an antibody titer higher than 40 U/mL (0.5%). The prevalence of aPL antibodies between the groups of placenta-mediated complications was similar (*p* = 0.17). In the subgroup analyses according to term vs. preterm labor, and LMWH treatment vs. no such treatment, there were no statistically significant differences between patients who tested negative and those found positive for aPL antibodies.

## 4. Discussion

We aimed to examine the prevalence of aPL antibodies in pregnancies with placenta-mediated complications >34 weeks of gestation. Our key findings were: (1) The prevalence of aPL antibodies in the study cohort was 4.9%. (2) The rates of positive aPL antibodies were comparable between the different subgroups of SGA, PET with severe features, and placental abruption. (3) The prevalence of aPL antibodies was similar between patients who delivered in the late preterm period and those who delivered at term.

The pathogenesis of APS-related pregnancy morbidity is considered to be placenta-mediated. Histological studies of aPL antibody-positive women found that the common features were placental infarction, impaired spiral artery remodeling, decidual inflammation, and the deposition of complement split products [21]. These pathological manifestations suggest the role of thrombotic, antiangiogenic, and inflammatory factors in the pathological process of the disease [7]. The antibodies affect numerous cellular processes, including blastocyst implantation in the endometrium; subsequent trophoblast proliferation, migration, and differentiation; and, eventually, antiangiogenic and prothrombotic activation, leading to placental insufficiency [21].

The prevalence of aPL antibodies in our cohort of patients diagnosed with various manifestations of placenta-mediated complications and delivering after 34 weeks of gestation is similar to the reported prevalence of aPL antibodies in the general obstetric population, ranging between 1.4 and 7% [16,22,23,24], and in the general population of women who had never conceived (3%) [22]. These results should be interpreted with caution because some prior studies did not examine anti-β2GPI antibodies [22,23], used different methods than recommended nowadays to evaluate for the presence of LAC [22,23], and consisted of a relatively small number of patients (<150 patients) [16]. Notably, when examining the prevalence of aPL antibodies in patients with prior venous thromboembolism (VTE), de Groot et al. found a 3.4% positivity rate of anti-β2GPI antibodies in the healthy control group [25].

Previous studies examining the prevalence of aPL antibodies in a population with clinical OAPS described a higher prevalence than found in our cohort, ranging between 9.6 and 36.6% [15,16,24]. This high variability could be explained by a small number of cases in some of the studies (112–148 patients) [15,16], using a lower antibody titer threshold than recommended for a positive aPL antibodies diagnosis [15], and not testing for LAC [24]. Moreover, as the clinical criteria of OAPS include placenta-mediated complications with delivery at an early GA, it is expected that the rate of aPL antibodies would be higher in those cases.

In a large retrospective analysis including 120 studies examining the prevalence of aPL antibodies in patients with clinical criteria for APS [26], aPL antibodies were present in approximately 6% of patients with pregnancy morbidity, in 13.5% of patients with stroke, in 11% with myocardial infarction, and in 10% of patients with deep venous thrombosis. This analysis has several limitations. For instance, only 11% of the studies performed all 3 criteria tests for aPL antibodies, and 36% of the studies used a low-titer aCL cutoff. They concluded their analysis by recommending appropriately designed population studies to examine aPL antibodies prevalence and associated events.

The prevalence of aPL antibodies in patients with obstetrical complications not meeting the revised Sydney criteria is much less studied. A previous study exploring the prevalence of aPL antibodies in late-onset pregnancy complications (>28 gestational weeks) in 100 patients found a prevalence of 31% [11]. However, they also included extra-criteria aPL antibodies such as β2GPI-Domain 1, immunoglobulin A (IgA) isotypes, and phosphatidylserine-prothrombin antibodies that are not part of the laboratory criteria for APS [3]. If including only criteria aPL antibodies, the prevalence rate decreased to 14%, which is still higher than we found in our cohort. This could be further explained by the fact that they also included stillbirth and placenta-mediated complications occurring < 34 weeks of gestation, which are APS criteria and may contribute to an overestimation of aPL antibodies prevalence.

In our study, we limited our cohort to significant placenta-mediated complications in late preterm and term pregnancies, while omitting less severe placenta-mediated complications such as PET without severe features and SGA neonates above the 5th percentile. We did not find a difference in the prevalence of aPL antibodies among the three subgroups in our study, albeit the placental abruption and PET with severe features groups comprised only 23 and 30 patients, respectively.

Fetal growth restriction (FGR) is one of the major causes of neonatal morbidity and mortality [27], with differing definitions in the literature, including estimated fetal weight or birthweight below the 10th percentile, below the 5th percentile, or below the 3rd percentile according to GA [28,29]. A previous meta-analysis [26] found that the prevalence of aCL antibody in patients with FGR at any GA was 17%, but the data regarding the prevalence of aPL antibodies in FGR pregnancies after 34 weeks of gestation is lacking. We found an aPL antibodies positivity rate of 3.9% in the SGA subgroup of patients.

We found a prevalence of 3.3% of aPL antibodies in the subgroup of patients with PET with severe features. PET with severe features complicates between 0.6 and 1.2% of pregnancies in Western countries [30]. There is a paucity of data regarding the prevalence of aPL antibodies in patients suffering from this complication. A study examining the prevalence of positive aPL antibodies testing in patients suffering from PET with severe features or placental insufficiency <36 weeks of gestation found a rate of 11.5% [16], albeit they included patients suffering from pregnancy morbidity that was considered part of APS and morbidity not considered part of the syndrome. A meta-analysis examining the association between aCL and pregnancies complicated by PET [31], found an odds ratio (OR) for aCL and PET of 2.86, and for PET with severe features, an OR of 11.15. Additionally, another study found that aPL antibodies positivity increased the risk for PET with severe features with an OR of 3.8 compared to controls [32].

Placental abruption is one of the major causes of perinatal morbidity, occurring in 0.4–1% of pregnancies [33]. Findings about the relationship between thrombophilia and placental abruption have shown conflicting results. While inherited thrombophilia was found to be associated with placental abruption [34], a meta-analysis did not find an association between aPL antibodies and placental abruption [35]. Data regarding the association between APS and placental abruption is limited, and to date, there are no studies examining the prevalence of aPL antibodies in late preterm and term placental abruption. We found a prevalence of 13% of aPL antibodies in this subgroup, although the relatively small number of patients suffering from this complication limited the ability to reach a definitive conclusion regarding this subgroup.

One of the largest studies in the literature regarding non-criteria obstetric APS examined 1000 patients with OAPS, and 640 patients with non-criteria obstetric APS [13] and found significant clinical and laboratory differences between the two groups. In the non-criteria obstetric APS, the rate of positive aPL antibodies was 82%, although one cannot compare this high prevalence to our results since their cohort was comprised mostly of patients who had a positive test for aPL antibodies who did not meet the clinical criteria for APS, and included a myriad of clinical criteria (such as preterm birth between 34 and 37 weeks without any apparent placental cause, repeated implantation failure, placental hematoma, and 1–2 consecutive miscarriages <10 gestational weeks). Limiting our cohort specifically to patients with pre-specified placenta-mediated complications, and then examining aPL antibodies prevalence in this cohort is the main novelty of our study compared to the one described.

There are several plausible explanations for the relatively low prevalence of aPL antibodies in our cohort. First, we excluded cases of active HBV, HCV, or HIV infection, which can result in a false-positive aPL antibodies result [36]. Another plausible explanation is that in our cohort, there were no patients with an SLE diagnosis, which is known to have a positive aPL antibodies rate ranging between 30 and 40% [37]. Lastly, we used strict laboratory criteria as recommended in the revised Sapporo criteria [3], while other studies also included low-to-medium aCL and anti-β2GPI antibodies levels cut-offs to be considered as a positive result [15].

Testing aPL antibodies in patients treated with anticoagulation medications has been studied extensively [38,39,40,41,42]. Previous studies showed conflicting results regarding testing for aPL antibodies in this subset of patients; while some recommended not testing under these medications [40,41], others stated it is possible under specific conditions [38,42]. The studies recommending against testing under anticoagulation mainly studied oral medications, while in our cohort, all patients received LMWH injections. Furthermore, a committee of the International Society on Thrombosis and Haemostasis [39] concluded that LAC tests are less affected by LMWH than by unfractionated heparin. The aPL antibodies tests drawn in our study in patients taking LMWH were drawn before the next dose of medications, and the laboratory received clinical information regarding anticoagulation use. Furthermore, we performed a subgroup analysis comparing the prevalence of positive aPL antibodies in patients taking LMWH and those who did not and did not find any statistically significant difference between the groups (6% vs. 2.7%, *p* = 0.12, respectively).

The aPL antibodies testing in our study was performed in the third trimester of pregnancy or in the immediate postpartum period while the patients were admitted to the postpartum ward. There may be great importance in testing patients in this time period since the postpartum period, and especially the first few weeks postpartum, confers the highest risk for VTE events [43,44]. Currently, guidelines do not recommend treating patients positive for aPL antibodies in late-term and term placenta-mediated complications [3], but if future studies show that they are at greater risk for thromboembolism, then testing them in the peripartum period could result in prompt anticoagulation treatment for those testing positive. Another advantage of testing patients while they are still admitted is a higher compliance rate. A previous study showed that only 50.9% of individuals with gestational diabetes mellitus had postpartum primary care follow-up [45]. Testing patients before being discharged home may potentially increase compliance and diagnose patients at higher risk for VTE events and future pregnancy morbidity.

Regarding the effect of pregnancy on aPL antibody testing results, a previous study examined the aPL antibodies variance during pregnancy [46]. They found that aPL antibody levels decreased marginally throughout pregnancy, and only 4% of the patients who tested positive for aPL antibodies at the screening test had a negative test in pregnancy. Therefore, we believe that the advantages of testing pregnant patients outweigh the minimal risk of receiving a false negative result.

Our study has several limitations. First, the patients in our study were tested only once, without a repeat antibody test 12 weeks after the initial one, as recommended [3]. Secondly, most of the patients in our study were in the SGA subgroup, while placental abruption and PET with severe features comprised 53 patients, limiting the ability to reach definitive conclusions regarding the prevalence in these specific subgroups. Lastly, our study did not have a control group of uncomplicated pregnancies.

Our study has numerous strengths. First, we included only a selective group of patients suffering from predefined severe late preterm and term placenta-mediated complications. Furthermore, we excluded pregnancies with abnormal genetic testing and abnormal findings on ultrasound scans. Notably, more than half of the patients in our study had genetic testing during pregnancy. Secondly, we excluded patients with active viral infections such as HIV and HCV, which could result in false positive aPL antibody results. Furthermore, we tested for all three antibodies related to APS, while the only study to date examining a similar group of patients did not [11]. Additionally, all the tests were performed in the same laboratory in a large tertiary center, adding to the credibility of the results. Lastly, this is one of the largest trials to date examining aPL antibodies prevalence in placenta-mediated complications not meeting APS clinical criteria.

It is well-known that treating patients with APLA syndrome with anti-thrombotic medications reduces maternal and neonatal complications [8]. The clinical utility of testing patients not meeting APS clinical criteria for aPL antibodies is still unknown. In our study, we described the prevalence of positive apL testing in this subset of patients. Whether treating these patients with anti-thrombotic treatment would alter their course of pregnancy and result in decreased perinatal complications is yet to be determined. Another issue to consider is the cost-effectiveness of testing.

In conclusion, the prevalence of positive aPL antibodies in patients suffering from placenta-mediated complications in late preterm and term pregnancies was 4.9%, lower than previously described [11,13]. Future large prospective studies will need to delineate whether patients who tested positive will gain from anticoagulative and anti-thrombotic treatment in future pregnancies, in order to decrease risks for recurrent maternal and neonatal complications.

## Figures and Tables

**Table 1:** Baseline maternal characteristics (*n* = 431).

Maternal age (years)	32.7 (±5.1)
Pre-pregnancy BMI (kg/m^2^)	22.2 (±3.9)
Obesity (BMI > 30 kg/m^2^)	22 (5.1%)
Smoking	32 (7.4%)
Chronic hypertension	3 (0.7%)
DM Type 1 DM Type 2 DM	5 (1.2%) 3 (0.7%) 2 (0.5%)
Autoimmune disease	6 (1.4%)
SLE	0 (0%)
Hypothyroidism	21 (4.9%)
Inherited thrombophilia	8 (1.9%)
Asthma	12 (2.8%)

Continuous variables are presented as mean (±SD), and categorical variables are presented as *n* (%). Abbreviations: BMI—body mass index; DM—diabetes mellitus; SLE—systemic lupus erythematosus.

**Table 2:** Pregnancy, delivery, and neonatal characteristics (*n* = 431).

Gestational age (weeks + days)	38 + 3 (±2 + 3)
Gravidity	1.9 (±1.4)
Parity 0 ≥1	0.5 (±0.9) 289 (67.1%) 142 (32.9%)
IVF	64 (14.9%)
Multiple pregnancy	11 (2.6%)
PTL (between 34 and 37 weeks)	68 (15.8%)
Aspirin treatment during pregnancy	40 (9.3%)
GDM	57 (13.2%)
Genetic testing in pregnancy cfDNA CMA WES	225 (52.2%) 43 (10%) 171 (39.7%) 11 (2.6%)
PET with severe features	31 (7.2%)
Placental abruption	23 (5.3%)
Mode of delivery SVD Instrumental delivery CD	199 (46.2%) 56 (13%) 176 (40.8%)
Neonatal birthweight (gram)* Birthweight < 2500 gr * Birthweight < 2000 gr *	2289.6 (±324.1) 331 (74.9%) 78 (17.7%)
SGA (≤10%) * SGA (≤5%) * SGA (≤3%) *	407 (92.1%) 400 (90.5%) 224 (50.7%)

* 442 neonates. Continuous variables are presented as mean (±SD), and categorical variables are presented as *n* (%). Abbreviations: IVF—in vitro fertilization; PTL—preterm labor; GDM—gestational diabetes mellitus; cfDNA—cell-free DNA; CMA—chromosomal microarray analysis; WES—whole exome sequencing; PET—preeclampsia; SVD—spontaneous vaginal delivery; CD—cesarean delivery; SGA—small-for-gestational-age.

**Table 3:** Antiphospholipid antibodies description (*n* = 431).

Variable	*n* (%)	*p*-Value
Prevalence of aPL Ab	21 (4.9%)	N/A
Test performed under LMWH	281 (65.2%)	N/A
Prevalence of specific Ab LAC aCL High titer (>40 U/mL) Anti-β2GPI High titer (>40 U/mL)	14 (3.2%) 6 (1.4%) 1 (0.2%) 6 (1.4%) 1 (0.2%)	0.08
More than 1 positive Ab	5 (1.2%)	N/A
Triple positive Ab	1 (0.2%)	N/A
Prevalence of aPL Ab SGA (≤5th) (*n* = 378) Severe PET (*n* = 30) Abruption (*n* = 23)	17 (3.9%) 1 (3.3%) 3 (13%)	0.17
Prevalence of aPL Ab Preterm labor (34–37 weeks) (*n* = 68) Term labor (>37 weeks) (*n* = 363)	4 (5.9%) 17 (4.7%)	0.67
Prevalence of aPL Ab according to LMWH Under LMWH treatment (*n* = 281) No LMWH treatment (*n* = 150)	17 (6%) 4 (2.7%)	0.12

Categorical variables are presented as *n* (%). Abbreviations: aPL—antiphospholipid; Ab—antibody; N/A—not applicable; LMWH—low molecular weight heparin; LAC—lupus anticoagulant; aCL—anticardiolipin; β2GPI—β2-glycoprotein-I; SGA—small-for-gestational-age; PET—preeclampsia.

## Data Availability

The data presented in this study are available on request from the corresponding author.
